# Willingness to pay for physician services at a primary contact in Ukraine: results of a contingent valuation study

**DOI:** 10.1186/1472-6963-13-208

**Published:** 2013-06-08

**Authors:** Andriy Danyliv, Milena Pavlova, Irena Gryga, Wim Groot

**Affiliations:** 1 School of Public Health, National University of ‘Kyiv-Mohyla Academy, Skovorody St. 2, 04655 Kiev, Ukraine; 2 Department of Health Services Research : Focusing on Chronic Care and Ageing, CAPHRI, Maastricht University, 6200 MD Maastricht, The Netherlands; 3 Topinstitute Evidence Based Education Research (TIER), Maastricht University, 6200 MD Maastricht, The Netherlands

**Keywords:** Willingness to pay, Demand, Contingent valuation, Physician services, Ability to pay, Payment acceptance

## Abstract

**Background:**

The existence of quasi-formal and informal payments in the Ukrainian health care system jeopardizes equity and creates barriers to access to proper care. Patient payment policies that better match patient preferences are necessary. We analyze the potential and feasibility of official patient charges for public health care services in Ukraine by studying the patterns of fee acceptability, ability and willingness to pay (WTP) for public health care among population groups.

**Methods:**

We use contingent valuation data collected from 303 respondents representative of the adult Ukrainian population. Three decision points were separated: objection to pay, inability to pay, and level of positive non-zero WTP. These decisions were studied for relations with quality profiles of the services, and socio-demographic characteristics of the respondents and their households.

**Results:**

The likelihood to object to pay is mostly determined by the quality characteristics of the services. Objection to pay is not related to corresponding behavior in real life. The likelihood of being unable to pay is associated with older age, lower income, and a larger share of household members with no income. The level of positive WTP is positively related to income (+7% per 1000 UAH increase in income) and is lower for people who visited a doctor but did not pay (−22%).

**Conclusions:**

Rather substantial WTP levels (between 0.9% and 1.9% of household income) for one visit to physician indicate a potential for official patient charges in Ukraine. User fees may cover a substantial share of personnel cost in the out-patient sector. The patterns of inability to pay support well designed exemption criteria based on age, income, and other aspects of economic status. The WTP patterns highlight the necessity for payments that are proportional to income. Other methodological and policy implications are discussed.

## Background

The Constitution of Ukraine guarantees free of charge provision of medical aid in public facilities for every citizen [1]. Thus, it practically bans all patient charges in the public sector. However, there is a mismatch between regulation and reality. This is caused by the quasi-formal charges (providers’ requests to pay official charitable contributions to the health care organization which should be voluntary in nature). There are also informal (under-the-table) charges in the form of cash or in-kind gifts paid to health care providers for better services. Such unregulated charges have distortive effects on healthcare provision and consumption [2-4]. Most important, they jeopardize equity in health care and create barriers to access even in the absence of official patient charges.

This ‘status quo’ inherited from the Semashko system established in Ukraine during the Soviet Union period, seems to currently satisfy only policy-makers who, as suggested by anecdotal evidence, receive healthcare abroad or in the best public facilities [5]. The political establishment guards zealously the tax-funded free-of-charge principle of public health care provision for the sake of retaining electoral support. However, this policy conservatism is at odds with the inefficiency of resource allocation and the high level of patient dissatisfaction with the healthcare system [6]. Ukrainian patients suffer from a lack of access to proper care (especially in rural areas) [7], long waiting lines, the reluctance of unmotivated medical staff to offer adequate care [2], and obsolete and inefficient treatment methods [8]. They are left with the option to seek better access and quality by means of quasi-formal and informal payments, unless they can use their personal connections to obtain the services they desire [2,9].

The data suggest that patients in Ukraine cover a substantial part of health care expenditure out of pocket. WHO data indicate that private out-of-pocket expenditure in Ukraine amounts to around 40.5% of total health expenditures [10] with a very small part being administered in the private sector [3] although the exact size of out-of-pocket payments in the private sector is unknown. This is one of the largest shares of out-of-pocket payments in Europe which contradicts the official ‘free-of-charge’ policy in Ukraine.

Most of the out-of-pocket spending is for medical goods. These should be provided to patients for free by the public health care settings, but patients have to purchase them outside the setting due to the absence of these medical goods in these facilities (see data at [10]). In addition to this, evidence suggests that the share of unofficial patient charges (i.e. quasi-formal and informal charges) at public health care settings is substantial [9,11,12]. Unregulated patient charges create a considerable financial burden on households and provoke reduced and unequal access to public health care services in Ukraine [2]. This problem is recognized at the national level (e.g. [7,8]). In view of this, it is not surprising that about 50% of the Ukrainian patients state that they either have to borrow money to pay for health care or to forgo services due to patient charges [9].

Obviously, there is a need for a more efficient patient payment policy. Together with other anticorruption measures, official patient charges may to a certain extent reduce the need to pay unofficial charges. They may provide extra revenues for health care providers, reduce inefficient health care provision and excess demand for health care [13]. However, the implementation of official patient charges is not straightforward. Apart from the need to establish a legal base for the implementation of such charges, the exact patient payment mechanism should be carefully designed and account for the population’s needs and ability to pay.

In this study, we analyze the potential and feasibility of official patient charges for public health care services in Ukraine. We address this issue by studying the patterns of fee acceptability, ability and willingness to pay (WTP) for public health care across population groups. The analysis is based on contingent valuation data from a household survey among a small representative sample of the Ukrainian population. Contingent valuation (CV) is a stated preference method that is widely applied in health services research to study individual willingness to pay for a health care good (for a review see e.g. [14-16]).

We focus on fees for physician services provided to patients with major health problems. We relate acceptability, ability and willingness to pay stated by the respondents to the specialization of the physician and to the quality/access profile of the physician. This is relevant for policy making because the role of primary care is still neglected in Ukraine as primary care providers are often bypassed by the patients and the number of general practitioners (GPs) is still insufficient. At the same time, the quality of and access to care is reported to be unsatisfactory [6], which is frequently explained by the severe under-financing of the system [17,18]. The unregulated patient payments are known to fill this financial gap. The question is whether Ukrainian patients are willing and able to pay officially for quality and access improvements. Our study can be a useful starting point for policy discussions in Ukraine as well as in other countries that face similar problems (especially other former socialist countries).

## Methods

Data were collected in a household survey among 303 respondents who agreed to participate in face-to-face interviews conducted in December 2009. An informed consent was obtained from all individuals included in the study. The study instruments and methodology were reviewed by the Institutional Review Board at the National University of Kyiv-Mohyla Academy and a waiver from a full ethical review was obtained. A stratified, multi-stage area probability sampling strategy was applied with 110 settlements as primary units and 270 voting precincts as secondary units. The sample is representative of the adult Ukrainian (aged 18 and older) population. However, for logistic reasons, the data collection was performed within a larger household survey. Therefore, information about the non-response rate and, consequently, non-respondents’ characteristics is not available.

The questionnaire contained various parts, including parts that focus on the past use of medical services, consumer stated preferences, and household and personal characteristics of respondents. We present the results of the analysis of the stated preference data collected via four CV tasks. Respondents were asked to state if they would be willing to pay for a visit to a physician with quality and access characteristics expressed by four profiles. In case a respondent was willing to pay for a given physician profile, a combination of payment cards and open-ended questions was applied. First, the respondent selected a payment interval from the card, then, an exact amount within the interval (open-ended question). Payment cards enabled framing respondents’ answers (preventing overstatement), while exact values elicited by the open-ended questions served as a more precise indication of the maximum WTP level. If a respondent was not willing to pay, s/he was asked to state the reason. The exact wording of the CV tasks is presented in Additional file 1. The four valuation profiles were designed in a way to estimate two separate effects on the WTP for physician services: (i) the effect of a physician’s specialization (general practitioner or medical specialist), and (ii) the effect of quality/access improvements (namely the joint effect of improvements in the state of medical equipment, maintenance of the physician office, and the reduction in waiting time in front of the physician’s office from 45 to 10 minutes). The four valuation tasks and the effects on the WTP studied are summarized in Table 1.

**Table 1:** Physician profiles included in the CV tasks and the effects on WTP studied

Attributes *	**Physician profiles included in the CV tasks**				**Effects on WTP studied**
	**GP basic profile**	**GP improved profile**	**Specialist basic profile**	**Specialist improved profile**	
Physician’s specialization	0 = GP	0 = GP	1 = Specialist	1 = Specialist	Specialization
State of the medical equipment	0 = Outdated	1 = Modern	0 = Outdated	1 = Modern	Quality/access improvements
Maintenance of the physician’s office	0 = Old-looking	1 = Renovated	0 = Old-looking	1 = Renovated	
Waiting time in front of the office	45 min	10 min	45 min	10 min	

* Two attributes remained constant in all profiles: attitude of the medical staff = polite, and travel time to the physician’s office = 15 min.

The socio-demographic and household characteristics are summarized in Table 2. The sample does not differ substantially from the entire Ukrainian population except for a slight over-presentation of women and some specific age groups. To account for this slight overrepresentation, we include these and other socio-demographic characteristics in the analysis.

**Table 2:** Socio-demographic characteristics of the sample (303 respondents)

**Characteristic**	**Observations**	**Percentage of total**	**Percent of non-missing**	**Mean**	**(S.D.)**
Age					
aged 18-34	72	23.76%	23.76%	47.5	(16.2)
aged 35-54	127	41.91%	41.91%	valid n = 303	
aged 55+	104	34.32%	34.32%		
Sex					
male	98	32.34%	32.34%		
female	205	67.66%	67.66%		
Place of residence					
village	122	40.26%	40.26%		
town (20–100) or small city (100–500)	116	38.28%	38.28%		
big city (500+) or capital	65	21.45%	21.45%		
Education level					
lower than secondary	61	20.13%	20.20%		
secondary	174	57.43%	57.62%		
higher or degree	67	22.11%	22.19%		
(missing)	1	0.33%			
Health status					
absolutely sick to bad	49	16.17%	16.17%		
fair	139	45.87%	45.87%		
good to perfect	115	37.95%	37.95%		
Voluntary insurance policy					
no	287	94.72%	95.35%		
yes	14	4.62%	4.65%		
(missing)	2	0.66%			
Size of the household					
1 member	41	13.53%	13.53%	3.03	(1.56)
2 or more members	262	86.47%	86.47%	valid n = 303	
Number of children in the household					
no children	181	59.74%	60.33%	0.55	(0.84)
1 or more children	119	39.27%	39.67%	valid n = 300	
(missing)	3	0.99%			
Share of household members who do not work or earn					
less or one half of family not working	166	54.79%	54.97%	0.53	(0.35)
more than half of family not	136	44.88%	45.03%	valid n = 302	
(missing)	1	0.33%			
Income (descriptive)					
not sufficient	105	34.65%	35.71%		
meets the need	126	41.58%	42.86%		
allows saving	63	20.79%	21.43%		
(missing)	9	2.97%			
Income (level, UAH)					
1000 UAH or less	58	19.14%	21.72%	2 346.8	(1 781.1)
from 1001 to 2000 UAH	104	34.32%	38.95%	valid n = 267	
from 2001 to 4000 UAH	66	21.78%	24.72%		
4001 UAH and more	39	12.87%	14.61%		
(missing)	36	11.88%			
Experience in visiting and paying to a physician					
did not visit	55	18.15%	18.97%		
visited did not pay	101	33.33%	34.83%		
visited and paid	134	44.22%	46.21%		
(missing)	13	4.29%			
Total	303	100%	100%		

We calculated the proportion of respondents who were willing and unwilling to pay for physician services (with a specified reason for their unwillingness to pay – object to pay and/or inability to pay). We also estimated the mean WTP amount. For the purpose of comparison, we looked at three measures of mean WTP: (i) including all answers (both positive and zero WTP answers), (ii) excluding protest answers (i.e. statements of unwillingness to pay due to an objection to pay fees), and (iii) including only positive WTP values. From a methodology point of view, protest answers should be excluded from the analysis since those who just object to pay may not really place a zero value on the services under valuation and would be willing to pay in a real market [19]. It should be noted however that this issue is still debated.

**Figure 1:**
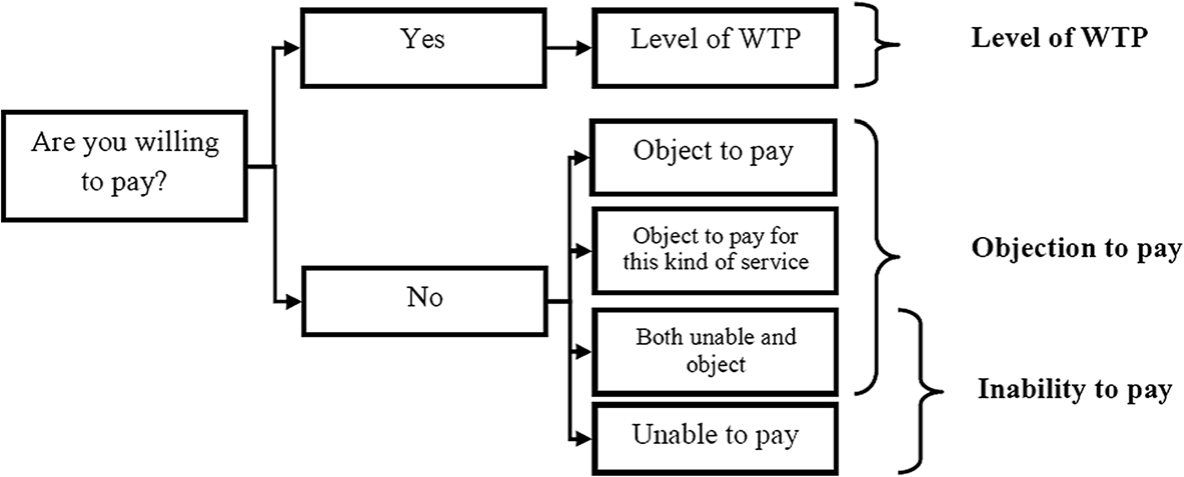
Decision sequence in the CV tasks and resulting modeling points.

We use a three-phase modeling approach because the WTP data were obtained using a sequence of three main questions (see Figure 1): first whether the respondent is WTP (positive or zero WTP), and second, given a positive WTP, what is the maximum WTP level, or given a zero WTP, what is the reason for the unwillingness to pay. Three main categories were used to structure the reasons for the unwillingness to pay: objection to pay, inability to pay or both. Hence, respondents who selected the last category were assigned as being both unable and objecting to pay.

Thus, as presented in Figure 1, three decisions were separated: objection to pay, inability to pay, and the level of positive non-zero WTP. For the first model (objection to pay), the cases (*y*=*1*) include those who stated that they “object to pay”, “object to pay for this kind of service”, or “both unable and object to pay”. They were compared to those who do not object (*y*=*0*), i.e. stated a zero WTP due to the inability to pay only, or a positive WTP. The second model (inability to pay) included those who stated that they either are “unable to pay” or “both unable and object” as cases (*y*=*1*). They were compared to those who are not unable to pay (*y*=*0*), i.e. stated a zero WTP due to objection only or a positive WTP. These two models have binary response variables; hence, we use random effect logistic regression (considering that one respondent evaluates four profiles). Denote the probability of a positive response (object to pay in the first model or being unable to pay in the second) for a respondent *i* valuing profile *j* as *P*_*ij*,*y*=1_. Then, the log odds can be described as a linear combination of the socio-demographic characteristics of the respondent and characteristics of the physician profile under valuation:

(1) $$\begin{array}{rl} \log\left(\frac{P_{\mathrm{ij},y=1}}{P_{\mathrm{ij},y=0}}\right) & =\log\left(\frac{P_{\mathrm{ij},y=1}}{1-P_{\mathrm{ij},y=1}}\right)\\ & =\beta_{X}X_{j}+\beta_{\mathrm{SDC}}\cdot \mathrm{SD}C_{i}+\beta_{0}+\epsilon_{\mathrm{ij}}+\nu_{i}, \end{array}$$

where *X*_*j*_ is a set of profile j specific characteristics, *SDC*_*i*_ are socio-demographic characteristics of the respondent *i*, *ϵ*_*ij*_ is a stochastic error term and *ν*_*i*_ is a respondent specific random element. In this specification exponential coefficients represent the odds ratios for the change in characteristics.

(2) $$\exp\left(\beta_{x*}\partial x\right)=\frac{p\left(Y|x*+\partial x\right)/\left(1-p\left(Y|x*+\partial x\right)\right)}{p\left(Y|x*\right)/\left(1-p\left(Y|x*\right)\right)}$$

The third model (level of WTP) included only positive WTP levels. However, the positive WTP distribution was found to be skewed compared to the normal distribution. Therefore, in the analysis, we use a logarithmic transformation of positive WTP, which better resembled a normal distribution. The logarithm of respondent’s *i* WTP for a visit to a physician with profile *j* can be specified as random effect linear regression (also considering that one respondent evaluated 4 profiles):

(3) $$\begin{array}{rl} \log\left(\mathrm{WT}P_{\mathrm{ij}}\right)= & \beta_{X}X_{j}+\beta_{\mathrm{SDC}}\cdot \mathrm{SD}C_{i}+\beta_{0}+\epsilon_{\mathrm{ij}}\\ & +\nu_{i}. \end{array}$$

In this specification, coefficients represent percent changes in WTP in response to a unit change in the characteristics:

(4) βX,SDC=∂logWTPij∂X,SDC=∂WTPijWTPij·∂X,SDC

A set of socio-demographic characteristics (see Table 2) was included in the three models, as well as an indicator of the physician’s specialization and an indicator of service improvement. The models were also checked for the inclusion of interactions between service characteristics and socio-demographic characteristics but this did not add to the model fit (hence, it reduced the model performance in terms of the Bayesian Information Criterion and degrees of freedom). Therefore, interactions were not included in the final models. The models were reduced to only statistically significant variables in order to see which factors had a stable effect. Both full and reduced versions are presented and discussed.

## Results

**Figure 2:**
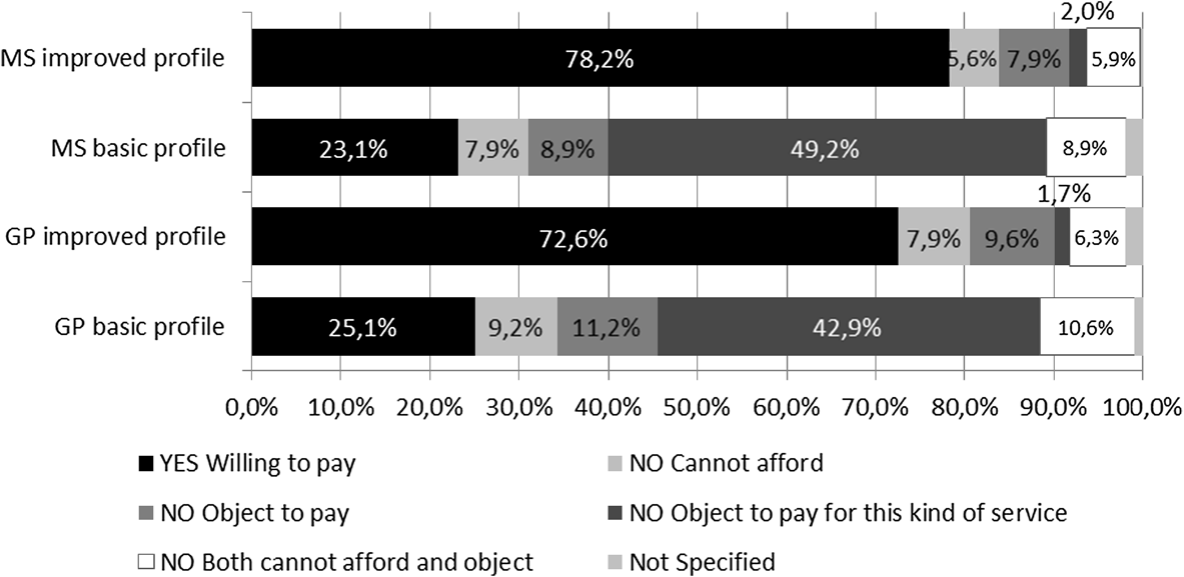
Proportion of respondents willing/unwilling to pay for physician services.

As displayed in Figure 2, a substantial part of the sample is willing to pay official fees for physician services. The share of respondents willing to pay is considerably higher (about three times higher) for physician profiles that indicate quality/access improvements than for the corresponding basic profiles. Specialization itself does not seem to have a large effect on the proportions of those who are willing or unwilling to pay. For profiles with less attractive quality/access characteristics, the dominant reason for being unwilling to pay is that people object to pay for such services. In contrast, profiles with better quality/access characteristics practically do not yield answers with such motivation for the unwillingness to pay. Other reasons for unwillingness to pay (i.e. cannot afford, object to pay for medical services, and both) are stated relatively infrequently. However, 11–20% of the respondents say that they are unable to pay (unable or both unable and object).

Table 3 presents the three estimates of the mean WTP for physician services: (i) when all answers are considered (both positive and zero WTP answers), (ii) when only non-protest answers (excluding those who object to pay) are considered, and (iii) when only positive WTP answers are considered. Regardless of the type of estimate, the mean WTP is significantly higher for both a GP and a medical specialist with better characteristics than for physicians with less attractive characteristics. Due to the large number of protest zeros for the less attractive profiles, the differences between the two mean estimates that include zero WTP answers (with and without protest answers) is substantial: 9.36 versus 20.39 UAH per visit for a GP (around 0.81 Euro and 1.80 Euro respectively), and 8.91 versus 22.10 UAH per visit for a medical specialist (around 0.77 Euro and 1.91 Euro respectively). However, for the profiles with more attractive characteristics the difference between these two means is not so large (around 5 UAH for both specialization modes). Specialization itself appears to have a minor impact on mean WTP although in most cases, WTP for a medical specialist is higher than that for GP services.

**Table 3:** Mean WTP for physician services

	Willingness to pay, UAH*					
	**All answers included**		**Objection answers excluded**		**Only positive WTP included**	
	**Mean (S.D.)**	**N**	**Mean (S.D.)**	**N**	**Mean (S.D.)**	**N**
GP basic profile	9.36 (23.47)	302	20.79 (31.44)	136	37.21 (34.06)	76
GP improved profile	32.59 (45.12)	297	37.37 (46.43)	259	44.81 (47.46)	216
Specialist basic profile	8.91 (23.13)	300	22.10 (32.23)	121	38.20 (34.39)	70
Specialist improved profile	40.50 (48.92)	301	45.14 (49.58)	270	51.87 (49.75)	235

*Average daily exchange rate during the period of data collection is 11.5424 UAH / EUR.

Table 4 presents the results of the three modeling processes (as described in the previous section): objection to pay, inability to pay, and level of positive WTP. Each of the modeling stages contains two versions of the model: a full model with all predictors, and a reduced model with only significant predictors. Further, we only discuss conclusions about predictors that are insensitive to the specification of the model, i.e. significant in both the full and reduced models.

**Table 4:** Results of modeling WTP: objection to pay, inability to pay, and level of positive WTP

**Dependent variable**		**Objection to pay *Random effect logit***				**Inability to pay *Random effect logit***				**WTP level *Random effect linear***			
		**1= object, unable and object**				**1= unable, unable and object**				**LN (WTP Positive)**			
		**0= unable, WTP>0**				**0= object, WTP>0**							
		**Full model**		**Reduced model**		**Full model**		**Reduced model**		**Full model**		**Reduced model**	
		**Coeff.**	**(S.E.)**	**Coeff.**	**(S.E.)**	**Coeff.**	**(S.E.)**	**Coeff.**	**(S.E.)**	**Coeff.**	**(S.E.)**	**Coeff.**	**(S.E.)**
	Specialization of a physician: 0 = GP 1 = Specialist	0.185	(0.212)			−0.689*	(0.301)	−0.727*	(0.286)	0.143*	(0.034)	0.138*	(0.033)
	Quality/access characteristics: 0 = Basic characteristics	−4.411*	(0.360)	−4.681*	(0.359)	−1.210*	(0.316)	−1.120*	(0.296)	0.366*	(0.045)	0.362*	(0.045)
	1 = Improved characteristics												
Age													
	aged 18-34	−0.086	(0.560)			−0.584	(1.082)	−0.319	(0.912)	0.132	(0.134)		
	aged 35-54	ref.				ref.		ref.		ref.			
	aged 55+	−0.223	(0.591)			2.082*	(1.032)	1.633*	(0.731)	−0.156	(0.143)		
Sex													
	female	ref.				ref.				ref.			
	male	−0.506	(0.459)			−1.060	(0.831)			0.051	(0.109)		
Place of residence													
	village	ref.				ref.				ref.			
	town (to 100)/small city (to 500)	−0.894**	(0.476)			0.165	(0.771)			0.068	(0.114)		
	big city (500+) and capital	−0.528	(0.581)			1.406	(0.993)			0.001	(0.141)		
Education level													
	primary or no education	−0.281	(0.579)			0.354	(0.838)			−0.089	(0.145)	−0.198	(0.132)
	secondary education	ref.				ref.				ref.		ref.	
	higher education or sc. degree	−0.117	(0.537)			0.378	(0.928)			0.186	(0.126)	0.220**	(0.118)
Health status													
	absolutely sick to bad	0.278	(0.643)			0.964	(0.897)			0.135	(0.157)		
	fair	ref.				ref.				ref.			
	good to perfect	0.318	(0.532)			−0.989	(0.927)			−0.139	(0.128)		
Voluntary insurance policy													
	no voluntary insurance	ref.				ref.				ref.		ref.	
	has voluntary health insurance	−0.751	(0.975)			−33.337	(>3x10^5^)			0.535*	(0.216)	0.448*	(0.208)
Size of the house hold													
	1 member (alone)	ref.				ref.				ref.			
	2+ members	0.663	(0.690)			0.193	(1.021)			−0.147	(0.168)		
Number of children in the household													
	No children in the household	ref.				ref.				ref.			
	1+ children in the household	0.616	(0.491)			1.713**	(0.956)			−0.044	(0.116)		
Share of household members who do not earn													
	<= half of household not working	ref.		ref.		ref.		ref.		ref.			
	> half of household not working	−1.102*	(0.488)	−1.039*	(0.425)	1.748*	(0.800)	2.561*	(0.685)	−0.016	(0.115)		
Income (descriptive)													
	not sufficient	ref.		ref.		ref.		ref.		ref.			
	meets the need	−0.521	(0.493)	−0.695	(0.468)	−2.402*	(0.827)	−2.505*	(0.697)	0.115	(0.123)		
	allows saving	−0.979	(0.652)	−1.633*	(0.579)	−4.925*	(1.408)	−5.334*	(1.293)	0.059	(0.151)		
Experience in visiting and paying													
	did not visit	−0.263	(0.569)			0.684	(1.005)	0.393	(0.881)	0.032	(0.135)	0.009	(0.123)
	visited did not pay	−0.055	(0.472)			0.886	(0.752)	1.537*	(0.701)	−0.208**	(0.114)	−0.219*	(0.110)
	visited and paid	ref.				ref.		ref.		ref.		ref.	
Level of income, UAH		0.000	(0.000)			0.000	(0.000)			70.6*(32.2) x10^-6^		67.3*(26.2)x10^-6^	
Constant term		2.405*	(0.893)	2.545*	(0.462)	−3.931*	(1.397)	−4.331*	(0.812)	3.001*	(0.217)	2.945*	(0.115)
Residuals' statistics													
	/lnsig2u	1.968		2.148		2.630		2.703		-		-	
	sigma_u	2.675		2.927		3.725		3.863		0.649		0.644	
	sigma_e									0.364		0.361	
	rho	0.685		0.723		0.808		0.819		0.761		0.761	
Model fit													
	Number of obs		989		1150		989		1133		499		513
	Wald chi2		150.73		170.43		43.73		49		117.3		115.9
		Ll	−449.9	Ll	−518.8	Ll	−260.4	Ll	−295.5	R2 w-in	0.229	R2 w-in	0.232
		AIC	943.87	AIC	1049.7	AIC	564.8	AIC	612.9	btw	0.145	btw	0.119
		BIC	1051.6	BIC	1080	BIC	672.5	BIC	668.3	ov-all	0.146	ov-all	0.123

Significance level: * p< 0.05; ** 0.05≤ p ≤0.10; ref. – reference group.

Objection to pay for physician services is found to be strongly related to the quality/access characteristics of the profile. As can be seen from the significant negative coefficients, profiles with better quality/access characteristics have lower odds (80–100 lower chances) to object to pay. This is in line with the finding above that those who object to pay for physician services with less attractive quality/access characteristics constitute the larger share of the respondents who are unwilling to pay. The indicators of economic status also significantly impact the probability of objecting to pay for physician services. Respondents living in households where most members (or respondents themselves) do not earn an income are less likely to object to pay. On the other hand, a high perceived household income (one that allows savings) might also reduce the inclination to object, though this effect is significant only in the reduced version of the model. An unstable effect, which disappears when the model is reduced to significant factors only, is found for the place of residence with town or small city residents being less likely to object. Other socio-demographic factors demonstrate no effect on the likelihood of objecting to pay.

Inability to pay is related not only to the profile characteristics, but also to the specialization of a physician. The effect of the quality/access improvements is smaller compared to the model of objection to pay, but still highly significant, with more attractive profiles having 3 – 3.3 times lower odds to yield an inability-to-pay response. A similar effect is observed for the specialization of the physician but with a slightly lower magnitude: a medical specialist has a twice lower chance of yielding zero WTP due to inability to pay. Akin to objection to pay, inability to pay is significantly reduced by the increase in the share of household members who earn an income and by the perceived income. However, unlike the objection to pay, respondents from households with more nonearning members are more likely to report that they are unable to pay. Perceived income is also a very strong predictor of inability to pay irrespective of the model specification, with higher income levels being associated with lower chances to report inability to pay. It is also notable that people aged 55 or more are much more likely to report being unable to pay (odds are 5 to 8 times higher than for middle-aged). Also, people who visited a physician during the last 6 months but did not pay for this are more likely to report being unable to pay, though this effect is not stable.

The level of positive (nonzero) WTP, akin to ability to pay, is strongly and significantly related to both profile characteristics and specialization of the physician. Respondents report a higher WTP for a medical specialist (around 14% more than for a GP) and for more attractive quality/access profiles (around 36% higher). A strong and stable significant positive effect on WTP is observed for respondents with voluntary health insurance which is associated with a 45-53% higher WTP. The WTP level is related to the monetary income level but not to the perceived one. Each 1 000 UAH (approximately 87 Euro) increase in household income is associated with around 7% increase in the WTP level. Finally, experience in paying and visiting a physician has a significant and rather stable effect similar to the one for inability to pay. Respondents who visited a physician during the last 6 months and did not pay for this report a 20-22% lower WTP than those who paid.

## Discussion

The interpretation of the results should be done with some caution due to the limitations of the study. The results might be affected to some extent by the small sample size. Therefore, we focus our conclusions on strong main relations that are not sensitive to the model specification. Estimations based on bigger samples might show more detailed variations of acceptability, ability and willingness to pay for physician services across population groups.

Another limitation relates to the methodology. Contingent valuation is known to be subject to hypothetical bias [20]. That is respondents might not behave in the real world in the same way as they stated in a hypothetical experiment. However, some empirical studies have shown that open-ended CV (such as that used in our study) produces effect sizes that are rather comparable to real world WTP values (e.g. [21]). Nevertheless, we are not inclined to interpret the mean WTP as an indication of the possible service fee because this requires detailed analysis of demand behavior under different payment regimes. Our results should be interpreted in terms of the mere existence of the potential for patient copayments and the main value drivers for the patients. They may also serve as an indication of the societal benefits obtained through consumption of services of a given quality.

In the contingent valuation task respondents were presented with the scenario of an official fee. Therefore, their WTP statements might be affected by their attitudes towards formal and informal payment practices. Not all Ukrainians are positive about paying formally [2]. Formal charges are not part of the personal communication between patient and physician. Thus, they do not necessarily add to the coverage of personnel cost (i.e. physician’s income) and do not assure better quality (i.e. quality and access desired by the patient). Besides, they may be charged on top of the informal charges causing a double burden for the patient. Moreover, Ukrainians are well aware of the fact that the official salary rate in the health care sector (1 555 UAH in December 2009 or around 135 Euro) is one of the lowest compared to other sectors of the Ukrainian economy [22]. Patients in many cases may perceive informal payments as an act of solidarity and a necessary supplement to the miserable official salary of physicians [23]. Taking these perceptions into account we might expect that on average the true WTP level of the respondents is higher than the ones stated in the presented contingent valuation task due to lack of trust in official financing channels.

Our results demonstrate that official patient charges have potential in Ukraine. Even when faced with less attractive characteristics, Ukrainians express a rather substantial level of WTP, although less than a quarter of them are willing to pay. However, for physician services with improved quality/access characteristics, the share of those willing to pay is more than 70% with an average WTP of 44.8 UAH for a visit to GP (3.9 Euro) and 51.9 UAH for a medical specialist (4.5 Euro). There are no reliable estimates of the cost of health care services in Ukraine due to the existence of the public funding system where facilities are financed on a line-item budget principle regardless of the number of services provided. These stated WTP levels, however, are rather substantial in comparison to the average monthly salary in the health care sector. Taking into account that primary care specialists are among the low-income medical workers, the stated WTP on average appear comparable or even higher than the official personnel costs.

The introduction of co-payments in the public health sector may have various effects both in terms of consumption patterns and the official cost of the services. The effects on consumption should be subject of demand modeling studies. From the system and provider’s perspective, co-payments generate additional funds that could be redistributed to achieve different goals. Our results suggest that Ukrainians place high economic value on quality and access improvements. Thus, patient charges can only be implemented together with effective investment policies targeted at improving quality and access. The probability to object to pay for these services is mostly explained by low quality/access characteristics. Additionally, the likelihood of the ability to pay and the level of positive WTP are positively and strongly related to the quality/ access profile. Combined with the evidence that Ukrainians in general are not satisfied with the quality of care they receive [6] these findings underline the necessity of quality/access improvements in health care. A rough and conservative estimation (the difference of the mean WTP in Table 3, objection answers excluded) suggests that the social benefit gained from simple improvements in the state of medical equipment, maintenance of the physician office, and reduction of waiting time is 16.5 UAH per visit to a GP and 23.0 UAH per visit to a medical specialist (in December 2009 prices). This can be regarded as an indication of the investment potential, although more robust estimates based on larger samples may provide more precise indicators. In Ukraine, increasing quality and access can not only be realized through investments in training, capital, and organizational changes, but is also tightly related to personnel remuneration. Failure to satisfy physicians’ needs may provoke both resistance to official charges and double charges: formal on top of informal.

Our results demonstrate that among the zero WTP answers, protesters (the objection motive) are not driven by economic or social barriers. The negative relation with the share of nonworking members in the household only supports this idea: it indicates that the more members depend on one’s alimony, the more responsibility one has for else’s health and life. This might increase the value of health care service and, consequently, decrease the likelihood of objection to pay despite the (in)ability to pay. Moreover, reporting objection to pay does not necessarily lead to similar behavior in real life as it is not related to the payment experience in the year before the survey (i.e. chances of paying in real life are similar for those who object to pay and for those who do not object). Thus, both from a methodological and a policy perspective it is rational not to account for the preferences of pure ‘protesters’.

As for the ability/inability to pay and the level of WTP, objective socio-economic barriers, such as age and economic status, apply. The relations with household income also support the theoretical validity of the construction of the models. It is notable however that inability to pay is related to the perceived income level, while the level of WTP is related to monetary income. This shows that inability is a perceptional issue depending on the evaluation of one’s own income level, while the level of the WTP is defined by real monetary budgetary constraints. This suggests that different mechanisms underlie the two stages of the decision about the willingness to pay for the physician services and this should be accounted for in the WTP modeling.

The substantial share of population that is unable to pay for physician services (at least 11.5% for the medical specialist with attractive characteristics) is concerning. This is in line with the extensive discussions in the literature (e.g. [24,25]) that patient charges should be implemented together with exemption criteria related to age and income. To relate co-payment levels to the level of income might also help to reduce financial barriers to access although this is difficult to achieve in practice. A successful example is Bulgaria where patient charges are anchored on the minimum income in the country [26] although this does not eliminate barriers to access.

It is also worth mentioning that we observe a slight preference among Ukrainians for a direct referral to medical specialist and bypassing a GP. This is expressed through the higher likelihood of reporting inability to pay and the lower WTP level for the latter. However, this preference is practically non-existent for services with less favorable characteristics although a bit more explicated for services with more attractive quality/access characteristics. In the latter case, people are willing to sacrifice only around 7 – 8 UAH to bypass primary contact. This indicates that price signals (such as higher charges for specialists’ services without a referral) might still be necessary to discourage bypass practices if official patient charges are introduced. However, the variation of this preference across population groups should be studied in more detail in order to design an effective threshold for discouraging direct visits to a specialist without a referral.

To our knowledge the WTP for physician services in Ukraine has never been studied before. Thus, testing the external validity using other studies may only be done in relative terms. In our study WTP estimates range from 0.9% to 1.9% of household income when protest answers are excluded and from 1.6% to 2.2% when only positive values are considered. This is rather consistent for example with the results from Spain where WTP for physician/out-patient services represent 2% of household income [27].

## Conclusion

In this paper, we have analyzed the potential and feasibility of official patient charges for public health care services in Ukraine. We addressed this issue by studying the patterns of fee acceptability, ability and willingness to pay (WTP) for physician services across population groups. We found that acceptability/objection to pay is mostly related to quality/access characteristics of the services and is not to socio-economic characteristics. Hence, the protesters do not seem to attach an intrinsic ‘zero value’ to the services, and should be excluded from the estimations of WTP. At the same time, the inability to pay and the level of WTP are related to socio-economic factors.

Our results demonstrate that the potential of patient charges for physician services is promising as the level of WTP for physician services is substantial despite the quality/access profile of the services. However, if patient charges are implemented, the lower ability and willingness to pay among vulnerable groups should be addressed by well designed exemption criteria based on age and income and by anchoring co-payment levels on income.

Importantly, patient charges cannot be implemented without quality/access improvements in Ukraine. The social benefits that can be gained from quality improvements in medical equipment, maintenance and a reduction in waiting time (expressed through an increase in mean WTP) are rather substantial. Additionally, we find a rather weak (around 7 UAH per visit) monetary preference for direct referral. Thus, in the context of strengthening the role of primary care, differential patient charges for different service levels may be called for.

## Abbreviations

WTP: Willingness to pay; CV: Contingent valuation; GP: General practitioner; UAH: Ukrainian hryvnia (Ukrainian national currency).

## Competing interests

The authors declare that they have no competing interests.

## Authors’ contributions

All authors were in the work group responsible for the design of the study. AD, MP, and IG developed the questionnaire. Data analysis was performed by AD under supervision and with recommendations from MP and WG. AD drafted the manuscript. MP, WG and IG commented and corrected the manuscript. All authors have read and approved the final version of the manuscript.

## Pre-publication history

The pre-publication history for this paper can be accessed here:

http://www.biomedcentral.com/1472-6963/13/208/prepub

## Supplementary Material

Additional file 1 **This file contains Appendix A where the English wording of the contingent valuation task from the survey is presented.** The appendix shows one of the physician profiles offered to the participants as well as the set of questions used to establish the maximum amount of money that a participant was willing to pay for a given profile. Click here for file
