# Supplementary material for: Willingness to pay for physician services at a primary contact in Ukraine: results of a contingent valuation study
Source: BMC Health Serv Res. 2013 Jun 8;13:208. doi: 10.1186/1472-6963-13-208 (PMC3695783; doi:10.1186/1472-6963-13-208)
Supplement: Additional file 1 — This file contains Appendix A where the English wording of the contingent valuation task from the survey is presented. The appendix shows one of the physician profiles offered to the participants as well as the set of questions used to establish the maximum amount of money that a participant was willing to pay for a given profile. [file 1472-6963-13-208-S1.pdf]

Appendix A.

Example of the contingent valuation task

Next questions concern your willingness and ability to pay for medical services in the state-owned and communal facilities. We are interested in your personal opinion!

Imagine that patients should pay official fees for the physician services in state owned and communal facilities. These fees are gathered by local health administrations or to social insurance funds. Further these funds may be used to increase quality and access to health care. Please indicate your willingness and ability to pay such official charges for physician services with different quality and access characteristics.

Q1. Would you like to pay official fee for a visit to a physician with the following characteristics in case you have major symptoms*:

CARD

General practitioner;
Obsolete equipment; Bad maintenance of the premises; Polite attitude of the medical staff; 15 min of travelling; 45 min of waiting in front of the office

Yes	***go to Q3.***
No	
D/K	

Q2. What is the reason for your unwillingness to pay?
CARD

I cannot pay
I am against paying for the health care services
I object to pay for this level of servicesr
I both cannot and object to pay for the services
D/K

Q3. What is the maximum amount that you are willing to pay?
CARD

Up to 20 UAH.
Between 20 and 60 UAH
Between 60 and 100 UAH
More than 100 UAH.

Q4. How much exactly would you be willing to pay for a visit?

* Earlier in the questionnaire respondents were asked to give examples of the major symptoms that will make her/him think of going to a physician. The tentative explanation was: “Major symptoms are those that worry you or cause some discomfort, and you feel uncertain if they pass or not. In general, they make you thinking about going to a physician.”
